# The missed crossing vessel during open pyeloplasty: a potential advantage of the robot-assisted approach in children

**DOI:** 10.1007/s11701-024-02006-5

**Published:** 2024-07-16

**Authors:** Suhaib Abdulfattah, Laura Zirel, Sameer Mittal, Arun Srinivasan, Aseem R. Shukla

**Affiliations:** 1 Division of Urology, Children’s Hospital of Philadelphia, 3401 Civic Center Blvd, 34th Street and Civic Center Blvd., 337 Laurel Lane, Philadelphia, PA 19104 USA; 2 Division of Urology, North Estonia Medical Center, J. Sütiste Tee 19, Tallinn, Estonia; 3 Division of Urology, Perelman Center for Advanced Care, Hospital of the University of Pennsylvania, 3400 Civic Center Blvd, Philadelphia, PA USA

**Keywords:** Pediatrics, Crossing vessel, Minimally invasive surgery, Ureteropelvic junction obstruction, Pyeloplasty

## Abstract

**Objective:**

To investigate whether the panoramic view offered by robot-assisted laparoscopic pyeloplasty (RALP) reduces the likelihood of missing a crossing vessel compared to open pyeloplasty in cases where initial pyeloplasty fails.

**Methods:**

A single institution redo-pyeloplasty database was reviewed for children treated between January 2012 to July 2023. Clinical history, imaging and operative details were reviewed to identify the etiology for the redo procedure.

**Results:**

Cohort consisted of 45 patients undergoing a redo RALP during the study period. 29 of 45 patients had an initial open surgical approach, whereas 16 had an initial RALP. 10 patients were noted to have a missed crossing vessel on redo pyeloplasty – 9 had an initial open approach whereas 1 had an initial RALP (*p*<0.0001).

**Conclusions:**

RALP may reduce the risk of missing a crossing vessel due to the panoramic view of the surgical field intrinsic to an intraperitoneal RALP approach.

## Introduction

Anderson–Hynes dismembered pyeloplasty is the treatment of choice for ureteropelvic junction obstruction (UPJO) in children, with overall success rates higher than 90% [1–4]. Since the first description of laparoscopic pyeloplasty (LP) by Schuessler in 1993, LP has been shown to provide similar success rates to open pyeloplasty (OP) with the advantage of shorter hospital stays, reduced pain postoperatively, and better cosmetic outcomes [5–7]. However, the main limitation of laparoscopic surgery has been the difficulty in intracorporeal suturing and knotting, ergonomics, and a long learning curve [7, 8]. In 2002, Intuitive Surgical’s da Vinci robot platform (Intuitive Surgical, Inc., Sunnyvale, CA) was introduced and enabled surgeons to overcome many of those difficulties while demonstrating excellent and comparable outcomes in terms of efficacy and safety [8, 9]. However, to date, there is no consensus regarding the gold-standard approach for the pyeloplasty.

Recurrent UPJO is reported to have an incidence of 3–11% after primary pyeloplasty in the pediatric population [10]. The reason for failure of the first repair is often intrinsic narrowing, peripelvic fibrosis, high insertion of the ureter, and obstruction by a lower pole crossing vessel that was missed at the time of the initial surgery [11–13]. While a crossing vessel may be diagnosed by vascular phases of a magnetic resonance urogram (MRU), this study is not universally obtained prior to pyeloplasty [14].We hypothesized that the panoramic view of the surgical field conveyed by intraperitoneal laparoscopy during a laparoscopic or robot-assisted approach, reduces the risk of missing an extant crossing vessel, and these approaches convey an advantage over open pyeloplasty in reducing the role of that anomaly as a proximate cause of pyeloplasty failure.

## Methods

We reviewed our IRB approved prospectively maintained institutional minimally invasive surgery registry for robot-assisted laparoscopic pyeloplasty procedures performed at our institution from January 2012 to July 2023. Baseline demographics, intraoperative and perioperative details including etiology of obstruction were recorded.

**Fig. 1:**
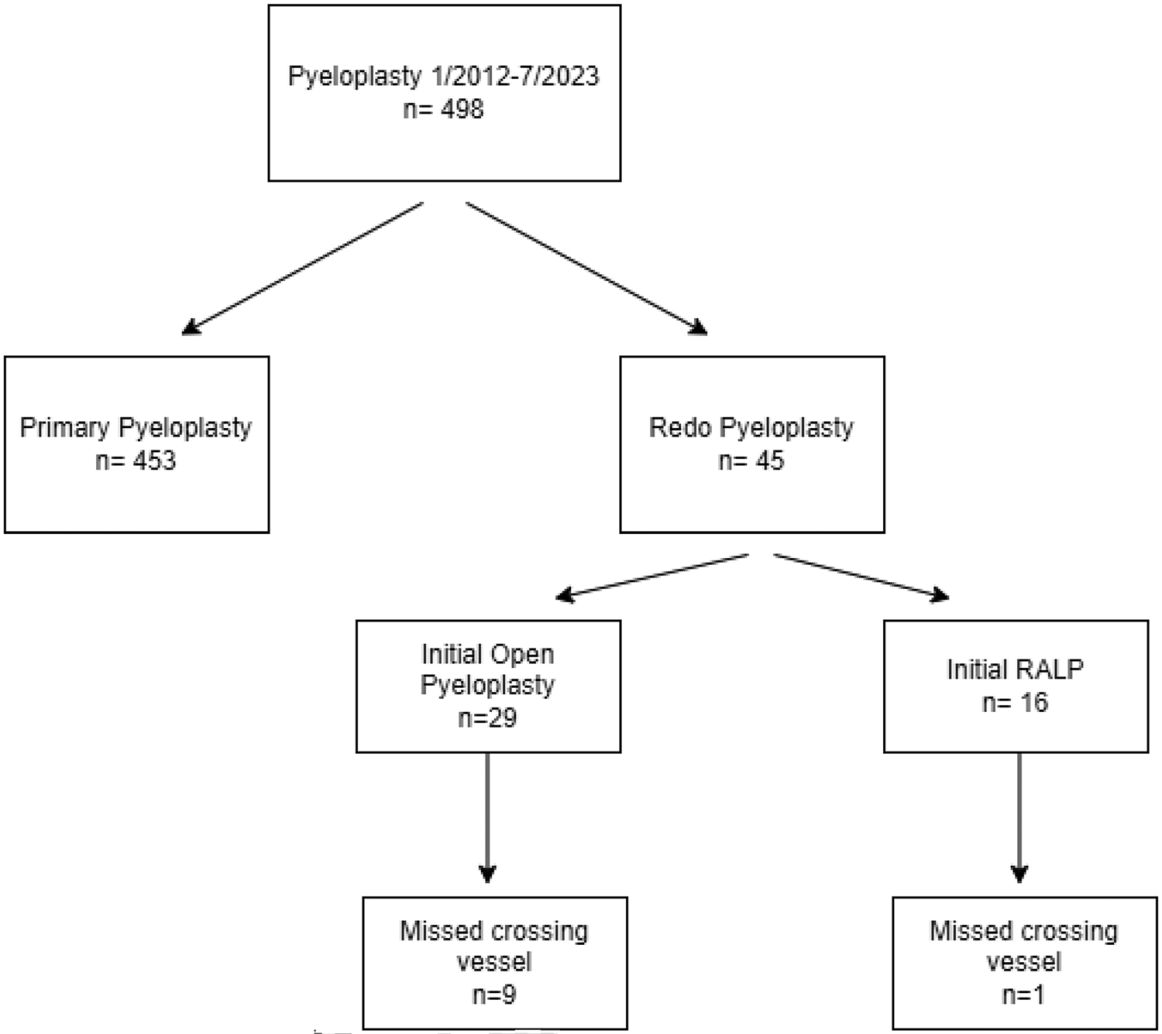
pyeloplasty and the missed crossing vessel

We stratified primary and redo pyeloplasty cases; redo cases were either robot-assisted laparoscopic pyeloplasty (RALP) or robot-assisted laparoscopic ureterocalicostomy (RALUC). In addition, we sub-stratified re-operative pyeloplasties based on initial surgical approach—whether the initial approach was open, laparoscopic or robot-assisted laparoscopic. All redo pyeloplasty procedures were done utilizing the Da Vinci Surgical System (Intuitive Surgical, Inc., Sunnyvale, CA). Figure 1 We reviewed operative notes and documented the cause of UPJ obstruction at time of primary and redo pyeloplasty in the redo pyeloplasty cohort.

The primary outcome we sought to define in this study was the incidence of a missed crossing vessel when a pyeloplasty fails and requires a redo repair. The secondary outcome was to assess whether the initial pyeloplasty surgical approach—open or RALP—correlated with a greater risk of missing a potentially obstructing crossing vessel.

Independent t test and Fisher’s exact test were used for continuous and categorical variables, respectively. *P* values were two sided and a *p* value < 0.05 was considered statistically significant. Analysis was done using the statistical software Stata BE 18.0 (Stata Corp, College Station, TX, USA).

## Results

Our initial cohort was comprised of 498 patients who underwent pyeloplasty between January 2012 and July 2023. Of these, 453 underwent successful primary pyeloplasty, while 45 underwent redo pyeloplasty. The median age for those undergoing successful primary pyeloplasty was 55.6 months (IQR: 16–139 months), while the age at primary unsuccessful pyeloplasty in the redo pyeloplasty cohort was much younger median age of 7 months (IQR: 4–71 months, *p* < 0.001).

In the redo pyeloplasty cohort, there was a median duration of 20 months (IQR: 11–48 months) between the primary and redo intervention, with the redo surgical intervention occurring at a median age of 56 months (IQR: 18–135 months). Of these 45 children undergoing redo surgery, 29 patients (64%) had undergone an initial open pyeloplasty, while 16 (36%) had an initial RALP. The etiology of obstruction at time of redo pyeloplasty—whether open or RALP, varied: 17 patients (38%) had a crossing vessel; 26 (58%) had intrinsic narrowing; and 2 (4%) had high insertion.

At time of redo pyeloplasty, 36 children (80%) underwent a repeat standard RALP as a salvage repair, while 9 (20%) underwent robot-assisted laparoscopic ureterocalicostomy (RALUC). Missed crossing vessels were identified as the etiology of pyeloplasty failure in 10 children (22%). A total of 9 of these children in whom a crossing vessel was not identified at time of pyeloplasty underwent an initial open pyeloplasty and 1 underwent an initial RALP (*p* < 0.0001). Notably, a review of the initial pyeloplasty operative note revealed that all ten missed crossing vessels were not seen by the operative surgeon and obstruction was ascribed to intrinsic narrowing without crossing vessel presence during their initial pyeloplasty procedure.

## Discussion

The incidence of persistent or recurrent UPJO is reported to be 3–11% after primary pyeloplasty in the pediatric population [10]. Generally, the reason for failure of the first repair is intrinsic narrowing, peripelvic fibrosis, high insertion of the ureter, and obstruction by the crossing vessel that was missed on the initial surgery [11–13].

The available options for managing failed pyeloplasty include balloon dilatation, antegrade and retrograde endopyelotomy, as well as redo pyeloplasty. Redo pyeloplasty can be performed through open surgery, laparoscopy, or with robotic assistance. There is no consensus regarding the gold-standard approach, though. However, the success rates for endoscopic interventions are relatively low as they range from 25 to 71% in secondary cases with complication rates as high as 14% [15, 16]. Therefore, re-operative pyeloplasty remains central to the management of recurrent UPJO in children [3, 17].

Crossing vessels are reported in 53–71% of patients with UPJO and in 19.2% of patients with a normal UPJ [18]. Richstone et al. [19] reported crossing vessels in 63.2% of patients who underwent laparoscopic pyeloplasty for primary pyeloplasty. They compared the histopathologic findings between those patients and the patients in whom UPJO was not related to extrinsic obstruction. They found that 43% of the patients with UPJO associated with a crossing vessel had no specific histopathologic changes referring the vessel as the sole etiology of obstruction. Therefore, they stated that crossing vessels do play role in the etiology of UPJO.

Rehman et al. [20] presented two cases of secondary UPJO in which crossing vessels were missed at retroperitoneal open pyeloplasty. Mittal et al. [13] reported 30 recurrent UPJO undergoing a secondary repair between 2012 and 2019. Eleven patients (36.7%) out of 30 had a persistent UPJO because of a missed crossing vessel on primary surgery.

Weiss et al. [14] reported on 166 pediatric patients with UPJO, wherein 78 patients were found to have a crossing vessel intraoperatively, while 88 did not. Their findings highlighted that increased age and the presence of pain at presentation predict the likelihood of a crossing vessel, while antenatal diagnosis does not reliably indicate its presence. Notably, despite antenatal hydronephrosis diagnosis, 25.6% of patients still had a crossing vessel, emphasizing the need for thorough evaluation in all pyeloplasty cases, especially when considering a shift from dorsal lumbotomy to a more anterior approach.

In a separate study, Wong et al. [21] conducted a 10-year retrospective study focusing on surgical management of hydronephrosis in children, particularly addressing crossing vessels. They observed a significant difference in median age at surgery between patients with and without crossing vessels (63.9 vs. 15.7 months, *p* < 0.001). Another study [14] noted a median age at surgery of 8 years for patients with crossing vessels compared to 0.95 years for those without, suggesting that symptoms related to crossing vessels manifest later in life, potentially leading to underdiagnosis during early pyeloplasty.

The aforementioned study demonstrates that the presence of a crossing vessel does not imply obstruction at diagnosis—since they may not become symptomatic until the child grows and urine production increases. Robotic-assisted laparoscopic pyeloplasty (RALP) offers the advantage of early—even when asymptomatic– detection and correction of crossing vessels by providing a panoramic view of the renal hilum and vessels. This correlates with our findings as those who had a primary unsuccessful pyeloplasty were much younger compared to those who were successful at the time of primary pyeloplasty procedure.

Regarding the choice of incision during open surgery, there has been debate, with flank incision preferred over dorsal lumbotomy due to better visualization of the renal hilum and vessels. Braga et al. [3] reported a higher risk of recurrent obstruction with dorsal lumbotomy compared to flank incision (8.1% vs. 3.1%). However, our study revealed that even patients initially treated with open pyeloplasty via flank incision later required redo RALP due to missed crossing vessels.

This study presents several limitations. Firstly, there is a lack of numerators for all successful pyeloplasties before referral for failure, which may impact the comprehensiveness of our analysis. Secondly, the retrospective design introduces inherent risks of selection bias, as well as the possibility of inconsistencies or missing details in the recorded data. In addition, as a single-institution study, our findings may not be broadly applicable limiting the generalizability of our findings.

## Conclusion

The management of recurrent UPJO presents a complex challenge, with many factors contributing to failure of primary pyeloplasty. Our study highlights that when undertaking a redo pyeloplasty, a missed crossing vessel will be the etiology in 22% of cases—and this occurs more commonly after open pyeloplasties. RALP may reduce the risk of missing a crossing vessel due to the panoramic view of the surgical field intrinsic to an intraperitoneal RALP approach.

## Data Availability

No datasets were generated or analyzed during the current study.
